# Author Correction: Ultrafast light-activated polymeric nanomotors

**DOI:** 10.1038/s41467-024-54179-0

**Published:** 2024-11-11

**Authors:** Jianhong Wang, Hanglong Wu, Xiaowei Zhu, Robby Zwolsman, Stijn R. J. Hofstraat, Yudong Li, Yingtong Luo, Rick R. M. Joosten, Heiner Friedrich, Shoupeng Cao, Loai K. E. A. Abdelmohsen, Jingxin Shao, Jan C. M. van Hest

**Affiliations:** 1 Bio-Organic Chemistry, Departments of Biomedical Engineering and Chemical Engineering & Chemistry, Institute for Complex Molecular Systems, Eindhoven University of Technology, 5600 MB Eindhoven, The Netherlands; 2 School of Aeronautic Science and Engineering, Beihang University, Beijing, 100191 China; 3 Laboratory of Chemical Biology, Department of Biomedical Engineering, Eindhoven University of Technology, 5600 MB Eindhoven, The Netherlands; 4 Laboratory of Physical Chemistry, Department of Chemical Engineering & Chemistry, Center for Multiscale Electron Microscopy and Institute for Complex Molecular Systems, Eindhoven University of Technology, 5600 MB Eindhoven, The Netherlands; 5 College of Polymer Science and Engineering, Sichuan University, Chengdu, 610065 PR China

**Keywords:** Molecular machines and motors, Self-assembly, Molecular self-assembly

Correction to: *Nature Communications* 10.1038/s41467-024-49217-w, published online 07 June 2024

In this article a reference was omitted from the ‘Introduction’ section. The sentence “First of all, the deposition of the gold component on the nanomotor chassis is difficult to control spatially.” should read “First of all, the deposition of the gold component on the nanomotor chassis is difficult to control spatially^42^. “

This has been corrected in the pdf version of the article.

[42] Toebes, B. J., Cao, F. & Wilson, D. A. Spatial control over catalyst positioning on biodegradable polymeric nanomotors. *Nat*. *Commun*. **10**, 5308 (2019).

