# Supplementary material for: Ultrafast light-activated polymeric nanomotors
Source: Nat Commun. 2024 Jun 7;15:4878. doi: 10.1038/s41467-024-49217-w (PMC11161643; doi:10.1038/s41467-024-49217-w)
Supplement: Supplementary file 1 — Supplementary Information [file 41467_2024_49217_MOESM1_ESM.pdf]

Supplementary information

Ultrafast Light-activated Polymeric Nanomotors

Jianhong Wang[a#], Hanglong Wu[a#], Xiaowei Zhu[b], Robby Zwolsman[c], Stijn R. J. Hofstraat[c], Yudong Li[a], Yingtong Luo[a], Rick R. M. Joosten[d], Heiner Friedrich[d], Shoupeng Cao[e], Loai K. E. A. Abdelmohsen[a], Jingxin Shao[a,*], and Jan C. M. van Hest[a,*]

[a] Bio-Organic Chemistry, Departments of Biomedical Engineering and Chemical Engineering & Chemistry, Institute for Complex Molecular Systems, Eindhoven University of Technology, 5600 MB Eindhoven, The Netherlands;

[b] School of Aeronautic Science and Engineering, Beihang University, Beijing 100191, China;

[c] Laboratory of Chemical Biology, Department of Biomedical Engineering, Eindhoven University of Technology, 5600 MB Eindhoven, The Netherlands;

[d] Laboratory of Physical Chemistry, Department of Chemical Engineering & Chemistry, Center for Multiscale Electron Microscopy and Institute for Complex Molecular Systems, Eindhoven University of Technology, 5600 MB Eindhoven, The Netherlands;

[e] College of Polymer Science and Engineering, Sichuan University, Chengdu 610065, P. R. China

[#]These authors contributed equally to this work.

* Corresponding author. E-mail addresses: J.Shao@tue.nl (J. Shao), and J.C.M.v.Hest@tue.nl (J. Hest)

Experimental

1. Materials

All materials were ordered from Sigma-Aldrich without further purification unless otherwise stated. Poly(ethylene glycol) methyl ether 1K, 2K and Boc-amino-poly(ethylene glycol) 3K were purchased from Rapp Polymers and freeze-dried before use. D,L-lactide (DLL) was purchased from Acros Organics. 1,4-Dioxane and tetrahydrofuran (THF) were obtained from Biosolve Chimie. Dialysis Membrane MWCO (12,000 - 14,000 Da) was supplied from Spectra/Pro®. Hoechst 33342, Wheat Germ Agglutinin, Alexa FluoTM 488 Conjugate, Dulbecco's modified eagle medium (DMEM), phosphate-buffered saline (PBS, pH 7.4), no mycoplasma fetal bovine serum (FBS), trypsin-EDTA, penicillin-streptomycin, and fluorescein isothiocyanate conjugate albumin from bovine serum (FITC-BSA) were purchased from ThermoFisher. Cyanine 5 labeled Small interfering RNA (cy5-siRNA) composed of sense (5'–ACCCUGAAGUUCAUCUGCACCACCG–3') and anti-sense (3'–ACUGGGACUUCAAGUAGACGUGGUGGC–5') strands was supplied by Integrated DNA Technologies. Doxorubicin (DOX) was purchased from MedKoo Biosciences. Ultrapure Milli-Q (Millipore) water (18.2 MΩ·cm) was used in this work.

2. Instruments

2.1 Nuclear Magnetic Resonance Spectroscopy (NMR): Routine proton nuclear magnetic resonance (^{1}H NMR) measurements were recorded on a Bruker AV 400 MHz Ultrashield^{TM} spectrometer, $CDCl_3$ was selected as the solvent and tetramethylsilane (TMS) as the internal standard for ^{1}H NMR.

2.2 Gel Permeation Chromatography (GPC): The molecular weights (Mw, Mn) and dispersity index (Đ) of the block polymers were measured using a Prominence-I GPC system (Shimadzu) with a PL gel 5 μm mixed D (Polymer Laboratories), equipped with a RID-20A differential refractive index detector. Polystyrene standards were used for calibration. THF was used as eluent, with a flow rate of 1 mL min^{-1}.

2.3 Dynamic Light scattering (DLS): The hydrodynamic size and dispersity index (PDI) of the nanoparticles were determined by a Malvern instruments Zetasizer (model Nano ZSP) equipped with a 633 nm He-Ne laser and avalanche photodiode detector. Zetasizer software was further used to analyze the data.

2.4 Scanning Electron Microscopy (SEM): Morphology of the nanoparticles was determined by a FEI Quanta 200 3D FEG.

2.5 Cryogenic transmission electron microscopy (cryo-TEM) and cryo-electron tomography (cryo-ET):

Data acquisition

Cryo-TEM and cryo-ET were performed on the TU/e CryoTITAN (Thermo Fisher Scientific) equipped with a field-emission gun operating at 300 kV, an autoloader station and a post-column Gatan energy filter. The TEM grids (R2/2, Cu, Quantifoil Jena grids, Quantifoil Micro Tools GmbH) were firstly plasma treated in a Cressington 208 carbon coater for 40 seconds before being used. Then, a 3 μL nanoparticle solution was pipetted onto the grid and blotted in a Vitrobot MARK IV (Thermo Fisher Scientific) at 100% humidity. Note that for cryo-ET sample preparation, 10 nm gold nanoparticle stock solution (nanoComposix, Inc), which were used as fiducial markers, was added into an aliquot of the dispersion before vitrification. The grid was then blotted for 3 seconds (blotting force: −3) and directly plunged and frozen in liquid ethane. Images were acquired via a post-GIF 2k Gatan CCD (charge-coupled device) camera.

Cryo-ET tilt-series acquisition was carried out using Inspect 3D software (Thermo Fisher Scientific). Prior to tomogram acquisition, dose series were first acquired up to ~ 70 $e^{-}\cdot Å^{-2}$, which showed negligible changes in the stomatocyte morphology. However, we found that some Au NPs attached to the outer

membrane of the stomatocyte can still move away from their original position due to the small deformation of the membrane induced by electron irradiation. As a result, an optimal electron flux of 0.9 $e^{-}\cdot Å^{-2}\cdot s^{-1}$ was used with an exposure time of 1.2 s/frame, resulting in a total dose of ~ 50 $e^{-}\cdot Å^{-2}$. Alignment of the tilt-series and tomographic reconstruction were performed in IMOD using the simultaneous iterative reconstruction technique (SIRT, 20 iterations). The parameters using in cryo-ET are shown below:

Angular sampling: -68° to 68° at 3° increments;

Magnification: 19000 ×;   Defocus: -2 μm;

Total image number: 46;   Total electron dose: ~ 1.0 $e^{-}.Å^{-2}$/frame.

Analysis of 3D Au NP distribution from tomographic data

Segmentation of Au NPs and quantitative 3D volume analysis (Supplementary Fig. 10-15) were performed in ImageJ/FIJI[1] and MATLAB (MathWorks). First, regions containing the Au stomatocyte were cropped and processed with a Gaussian blur (standard deviation = 1) in MATLAB. The data were then imported into the FIJI software and the Au NPs were segmented mainly using Otsu thresholding[2]. Note that the Au NPs from the small stomatocyte (Supplementary Video 6) attached to the studied large stomatocyte were not included in our analysis and were manually removed from the binary mask. After binarization, the volume and the centroid coordinates of all the Au NPs were obtained by performing 3D volume and 3D centroid analysis in FIJI. The rest of the data analysis was conducted in MATLAB using the following procedures: (1) The 3D centroid and volume data were imported into MATLAB and plotted using the MATLAB built-in function *scatter3*. (2) The 3D centroid matrix of Au NPs was rotated to a desired viewing angle using a rotation matrix from Ruler angles. (3) The centroids of the Au NPs inside the stomatocyte cavity were extracted and sorted along the z-axis. (4) The Au NP distribution at a given z-height was analyzed by generating a radial average profile from a 2D Au volume map (Supplementary Fig. 12). (5) The whole stomatocyte was divided into several segments along the z-axis and the average Au density was calculated in each segment. Note that it is of key importance to consider the boundary conditions when estimating the surface area of each segment of the stomatocyte. In particular, when calculating the Au density for the segment close to the boundary, which includes the cavity opening, it is essential to account not only for the lateral surface area but also for the contribution of the top surface area around the cavity opening. Neglecting this contribution would lead to an overestimation of the Au density around the cavity opening area. Therefore, we calculated the lateral surface area of all 40 segments, each with a reduced segment thickness of ~ 12 nm (Supplementary Fig. 15c), and separately estimated the top surface area as well as the Au density around the stomatocyte cavity opening (Supplementary Fig. 15d). The original data are plotted in Figure 5m and the averaged Au density distribution from five data points is shown in Supplementary Fig. 15d. Our results consistently demonstrate that the Au density at the bottom of the stomatocyte is higher than that at the opening.

Data visualization

Visualization of 3D Au NP distribution on Au-stomatocytes was carried out in Tomviz[3] and MATLAB (MathWorks)[4].

2.6 Two Photon-Confocal Laser Scanning Microscopy (TP-CLSM): Fluorescent images were recorded using a CLSM (Leica TCS SP8X) equipped with two-photon laser source (Chameleon Vision, Coherent, USA).

2.7 Microplate Reader: Cell viability was evaluated via a microplate reader (Safire[2], TECAN). The

reaction progress was monitored via the fluorescent signal from fluorescein on the Spark ® 10M microplate reader (TECAN).

2.8 UV-vis spectroscopy: UV-vis extinction spectra was determined by UV-vis spectroscopy (V-650, JASCO) using a 1 mL black quartz cuvette.

2.9 Infrared camera: IR thermal images of the solution (0.5 mL) during irradiation were acquired with a compact thermal imaging camera (FLIR E54) and quantified by FLIR tools software.

2.10 Thermometer: Temperature profiles of the solution (0.5 mL in a 1.5 mL Eppendorf tube) during irradiation were recorded with a thermometer (Chauvin Arnoux, C.A 1823).

2.11 NanoSight Tracking Analysis (NTA): Nanosight Tracking Analysis was performed on a Nanosight NS300 equipped with a laser channel (488 nm) and sCMOS camera. To analyze the autonomous motion under laser (660 nm) irradiation, the NanoSight was equipped with an Electron Multiplication Charge Coupled Device and external light source (660 nm, UltraLasers).

Movement analysis: NTA was used to study the motion of nanoparticles and calculate their mean squared displacement (MSD), following previously published procedures[5-7]. The motion of nanoparticles was analyzed by tracking both the X and Y coordinates of at least 30 particles for 30 seconds. MSD curves were extracted from NTA recorded trajectories using the following equation,

$$\mathrm{MSD} = \left[\Delta r^2(t)\right] = \left[\frac{1}{N}\sum_{i=0}^{N}\left(r_i(t)-r_i(0)\right)^2\right]$$

where r = radius, t = sampling time, and MSD(t) = 2dD. Here D = diffusion coefficient and d = dimensionality (NTA measurements have dimension d = 2). The equation MSD = $(4D)\Delta t + (v^2)(\Delta t^2)$ was used to fit the MSD curves. From the fitting of the MSD curves, the average particle velocity was extracted. According to the particle diffusion coefficient, as described by Galestonian's diffusiophoretic model, a particle undergoing Brownian motion displays a linear MSD fitting line over time with the slope determined by the diffusion coefficient $D = KB\ T/(6\pi\eta R)$. From this model, if the particles are in Brownian motion, the linear component of the MSD, according to the equation MSD = $(4D)\Delta t$, can be extracted. Based on the X and Y coordinates extracted from the videos, the mean squared displacement (MSD) was determined using a tailor-made Python script.

Code availability

The tailor-made Python script for motion analysis is available upon request.

3. Synthesis and methods

3.1 Preparation of gold nanoparticles (Au NPs)

In a 20 mL vial, poly(acrylic acid) (PAA, 0.8 mg) and $HAuCl_4.4H_2O$ (1.2 µL, 1 mg mL^{-1}) were added in 1 mL of Milli-Q water. The mixture was stirred at a rate of 200 rpm for 10 minutes at RT. Then, a solution of $NaBH_4$ (1 mL, 5 mM) was added dropwise and the reaction mixture was stirred for another 10 minutes at RT. The solution was centrifuged down (4600 g, 10 minutes), supernatant was removed and MilliQ water was added. This solution was washed until the supernatant was clear. Finally, the precipitate was dispersed in 1 mL Milli-Q. Samples were stored in the fridge for further use.

3.2 Motility studies

The autonomous motion of Au-stomatocytes and control nanoparticles was measured by the nanoparticle tracking analysis (NTA) technique with an external 660 nm laser (UltraLasers). Samples were suspended in 1 mL Milli-Q water and loaded in the NTA chamber using a syringe. Then, the nanoparticles'

movement was recorded for 30 s. The NTA 2.2 software was used to track and analyze the trajectories of single nanoparticles.

3.3 In vitro experiments

3.3.1 Cell culture

Breast cancer cells (4T1), human cervical cancer cells (HeLa) and mice fibroblast cells (NIH/3T3) were cultured in DMEM medium containing 10% FBS, 1% 100 U mL^{-1} penicillin & streptomycin in the cell incubator (ThermoFisher) in an atmosphere of 5% CO_2 at 37 °C and 70% humidity.

3.3.2 Cell viability evaluation of Au-stomatocytes

To evaluate the feasibility of the nanomotor for nanomedicine applications, we evaluated the in vitro cytotoxicity of the Au-stomatocytes via a standard CCK-8 assay using a microplate reader (Safire^2, TECAN). To measure the biocompatibility, cells were seeded in 96 well plates at a density of 1×10^4 cells per well with 100 µL of DMEM containing FBS, penicillin and streptomycin for each well. The cells were incubated overnight. Then, the medium of each well was refreshed with medium containing Au-stomatocytes at different concentrations (0, 20, 40, 60, 80, 100, 200, 400 µg mL^{-1}). After incubation for 24 h, the cells were washed three times with PBS and treated with 100 µL of DMEM containing 10% CCK-8 for each well for 2-4 h. The absorbance of each well at 450 nm was recorded by a microplate reader. For each group, triplicated wells were tested, and the data were presented as mean ± SD.

To investigate the cell viability upon laser irradiation, HeLa cells were seeded in a 96-well plate at a density of 1×10^4 cells per well and cultured overnight. The cells were further incubated with medium containing Au-stomatocytes at different concentrations (0, 50, 100, 200, 400, 800 µg mL^{-1}) for 4 h before laser irradiation (660 nm and 808 nm, 1 W, 5 min). The cells were thereafter cultured for 20 h and the cytotoxicity was then measured via CCK-8 assay.

3.3.3 Intracellular uptake of DOX loaded Au-stomatocyte nanomotors (DOX-Au-stomatocytes)

To evaluate the intracellular uptake behavior, HeLa cells were cultured in µ-Slide 8 wells at a density of 2 × 10^4 cells per well with 200 µL of DMEM. The cell nucleus and cell membrane were stained with Hoechst 33342 and Alexa Fluor™ 488. DOX-Au-stomatocytes (20 µL) were added to the cell medium immediately before a standardized time scanning imaging sequence under TP-NIR irradiation.

3.4 Fe (FEM) simulation of temperature gradient around a moving Au-stomatocyte

To understand the temperature distribution around a single Au-stomatocyte, we employed the COMSOL Multiphysics software package to carry out finite element method (FEM) simulations to analyze the heat and fluid flow around an Au-stomatocyte in motion under varying intensities of laser light. We modeled the nanoparticles as spheres with a uniform diameter of 5 nm and assumed minimal clustering, which has little effect on their optical behavior. For computational efficiency, we represented the Au-stomatocyte shape in a simplified two-dimensional axisymmetric configuration. The numerical setup is described in Supplementary Fig. 16. Our approach involved solving the fundamental conservation equations related to mass, momentum, and energy:

$$\nabla \mathbf{u} = 0$$

$$\rho \frac{\partial \mathbf{u}}{\partial t} + \rho(\mathbf{u}\cdot\nabla)\mathbf{u} = -\nabla p + \mu \nabla^2 \mathbf{u}$$

$$\rho C_p \frac{\partial T}{\partial t} + \rho C_p \mathbf{u}\cdot\nabla T = k\nabla^2 T + Q_{NP}$$

where $\boldsymbol{u}$ is the velocity vector, ∇p represents the pressure gradient, T denotes the temperature, Q_{NP} refers to the heat generation by NPs, and ρ, C_p, and μ symbolize the density, specific heat capacity, and viscosity

of the liquid, respectively.

The photothermal effect of Au-particles under laser exposure was assessed using Mie scattering theory. This theory predicts the absorption cross-section area S_{abs} of a spherical Au-particle with a radius of 5 nm ($r_{NP} = 5$ nm) to be 0.136 nm^2 at a 660 nm laser wavelength. The rate of energy absorption is calculated by the product of S_{abs} and the laser's irradiance I_s. The energy absorbed by the Au particles is entirely converted into heat, functioning as a heat source. In our study, we simulated the photothermal heat generation from these nanoparticles by modeling it as a surface heat flux spread along the circumference of the stomatocyte segments. A boundary heat flux was applied to the stomatocyte's surface, corresponding to the total heat generated by the nanoparticles per unit area within the segments. The computational domain's boundaries were set to a constant temperature of 303.15 K, equivalent to room temperature.

For fluid dynamics, a no-slip boundary condition was enforced on both inner and outer surfaces of the stomatocyte. Additionally, a zero-stress condition was applied at the domain's right boundary. The top of the domain was subjected to a Dirichlet velocity boundary condition, allowing for the adjustment of the stomatocyte's translational velocity. The governing equations, along with the specified boundary conditions, were solved numerically using the finite element method.

Our simulation results confirm the existence of a temperature gradient along the axial direction of the stomatocyte. The averaged temperature gradient ∇T with an output power of 1.5 W was calculated to be ~ 100 $\mu K\ \mu m^{-1}$ (Supplementary Table 6) using Equation (1):

$$\nabla T = \frac{\oiint_{\Gamma_{UW}} T ds/S_{UW} - \oiint_{\Gamma_{DW}} T ds/S_{DW}}{\oiint_{\Gamma_{UW}} z ds/S_{UW} - \oiint_{\Gamma_{DW}} z ds/S_{DW}} \quad (1)$$

where the S_{UW} and S_{DW} are the total surface area of Γ_{UW} and Γ_{DW}, respectively.

To further understand the propulsion forces needed to attain high motion speeds, we calculated the drag forces exerted on the stomatocyte at different speeds, as the propulsion forces are equivalent to drag forces under low Reynolds number conditions. Our analysis reveals that overcoming drag forces at a speed of 125 $\mu m\ s^{-1}$ only requires a propulsion force of ~ 0.5 pN (assuming the nanomotor is spherical) based on Stokes' law, or ~ 0.3 pN based on the solution of the full Navier–Stokes equations, considering the shape factor of the stomatocyte (Supplementary Table 6).

Importantly, our analysis reveals a linear relationship between the temperature gradient ∇T and the laser power input, and the calculated drag force (F_d) is also found to be almost linear with the temperature gradient (Supplementary Table 6 and Supplementary Fig. 17). This correlation can be mathematically expressed as $F_d = C\nabla T$, where C represents a constant coefficient. This finding strongly suggests that the propulsion mechanism of the Au-stomatocyte is primarily driven by the thermophoresis effect. Here it is crucial to emphasize again that the key factor enabling ultrafast speed in our system is the precise alignment of the temperature gradient with the motion direction, which also allows for highly directional motion control. It is also worth noting that the steady-state temperature around the stomatocyte can be rapidly achieved within a few milliseconds which is consistent with our observation of instantaneous nanomotor motion when activated by the laser irradiation.

4. Supplementary Figures and Tables

Supplementary Table 1. Summary of selected examples of nanomotor velocities and propulsion mechanisms

Nanomotor type	Maximum Velocity	Driving force	Reference
Semi-Yolk@Spiky-Shell Nanomotor	~6.06 μm s^{-1}	Thermophoretic force	9
Polydopamine nanomotors	14.9 μm s^{-1}	Thermophoretic force	10
CuS/Pt nanomotors	17.4 μm s^{-1}	O_2 and self-thermophoretic force	11
Light-driven nanorod motors	~28.16 μm s^{-1}	Thermophoretic force	12
Janus mesoporous silica motors	~47.73 μm s^{-1}	Self-thermophoretic force	13
Asymmetric Hydrogel Nanomotors	~48.75 μm s^{-1}	Thermophoretic force	14
Au nanomotors	50 μm s^{-1}	Acoustic streaming force	15
Mesoporous SiO_2/Au nanomotors	86 μm s^{-1}	Thermophoretic force	16
Light-driven nanomotor	125 μm s^{-1}	Thermophoretic force	This work
Self-Propelled Enzymatic Nanomotors	2.08 μm s^{-1}	Bubble	17
Fe^0 Nanomotors	20 μm s^{-1}	Bubble	18
Nitric Oxide-Driven Nanomotor	10.9 μm s^{-1}	Bubble	19
NO-Driven Nanomotors	3 μm s^{-1}	Bubble	20
Bienzymatic Spiky Janus Nanomotors	9.25 ± 1.71 μm s^{-1}	Bubble	21
Enzyme-Mediated	19.8 μm s^{-1}	Bubble	22
MnO_2-Based Nanomotors	5.8 μm s^{-1}	Bubble	23
Streamlined Mesoporous Silica Nanomotors	10.5 μm s^{-1}	Bubble	24
Glucose-Powered nanomotors	20.4 μm s^{-1}	Bubble	25
Carbonaceous Nanoflask Motors	30.34 μm s^{-1}	Bubble	26
Urease-Powered Polydopamine Nanomotors	10.67 μm s^{-1}	Bubble	27

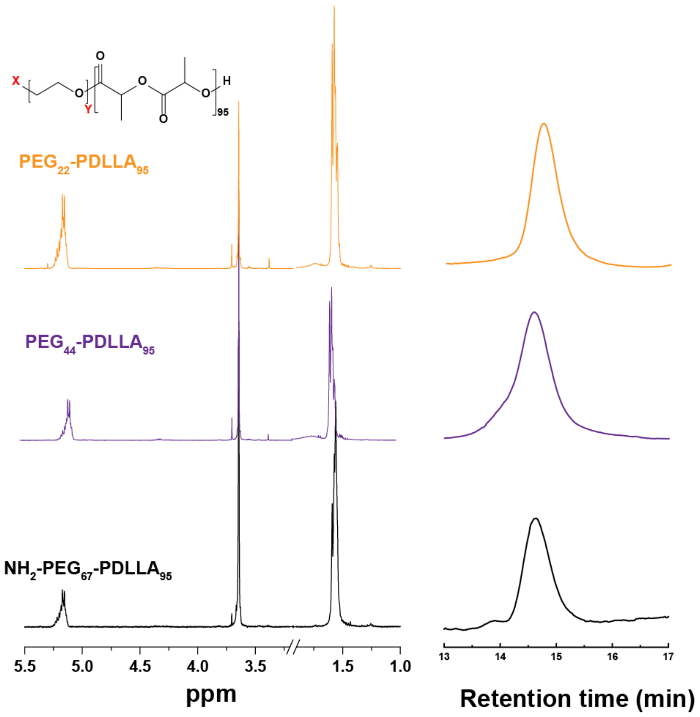

Supplementary Fig. 1. Characterization of PEG-PDLLA co-polymers. ^{1}H NMR spectra and GPC of PEG_{22}-$PDLLA_{95}$, PEG_{44}-$PDLLA_{95}$, NH_2-PEG_{67}-$PDLLA_{95}$.

Table S2. Overview of PEG-PDLLA block copolymers compositions. Degree of polymerization (DP) of copolymers is calculated by integration of characteristic NMR proton peaks.

Sample	DP (NMR)	M_W/kDa (GPC)	Đ (GPC)
PEG_{22}-$PDLLA_{95}$	90	13.6	1.08
PEG_{44}-$PDLLA_{95}$	91	14.4	1.09
NH_2-PEG_{67}-$PDLLA_{95}$	98	11.6	1.05

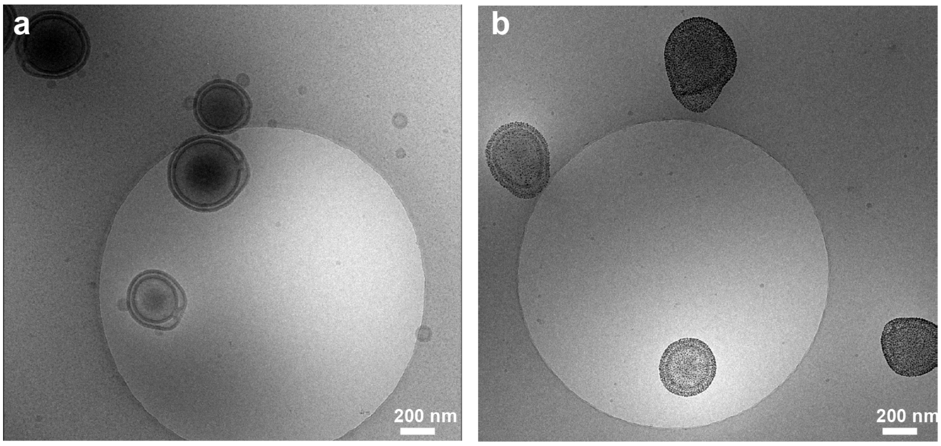

Supplementary Fig. 2. Cryo-TEM images of **a)** stomatocytes and **b)** Au-stomatocytes (10 experiments were repeated independently with similar results). All the scale bars = 200 nm.

Table S3. Zeta potential of stomatocytes and Au-stomatocytes.

Sample	Zeta potential (mV)
Stomatocytes	1.6 ± 0.8
Au-stomatocytes	-16.1 ± 1.1

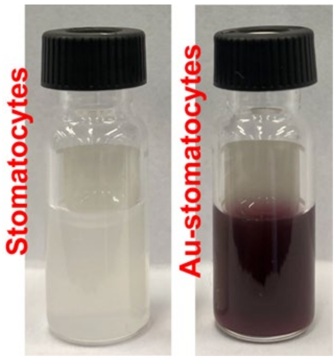

Supplementary Fig. 3. Photographs of uncoated stomatocytes and Au-stomatocytes in Milli-Q.

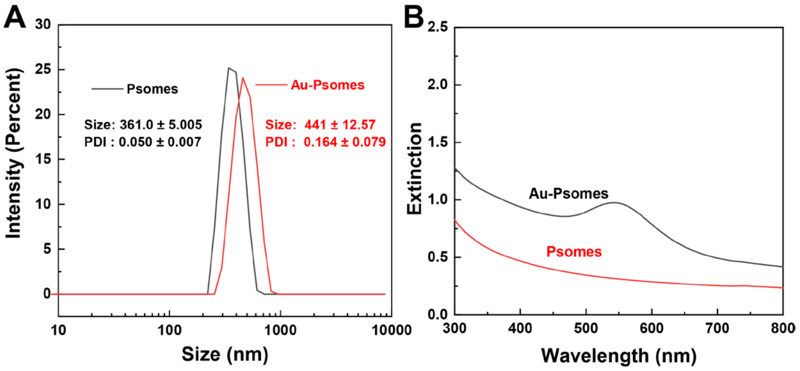

Supplementary Fig. 4. **a)** DLS data and **b)** UV-vis extinction spectra of polymersomes and Au-polymersomes.

Table S4. Output laser power and corresponding power density.

Output laser power (W)	Intensity (mW cm^{-2})
0.75	~1500
1	~2000
1.1	~2200
1.2	~2400
1.3	~2600
1.4	~2800
1.5	~3000

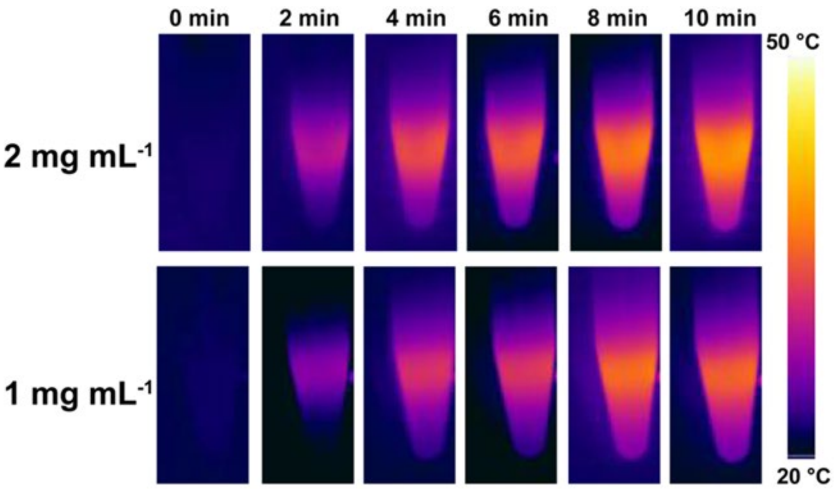

Supplementary Fig. 5. Temperature change images of Au-stomatocytes at different concentrations (1 mg mL^{-1} and 2 mg mL^{-1}) upon laser irradiation (1.5 W, 10 min).

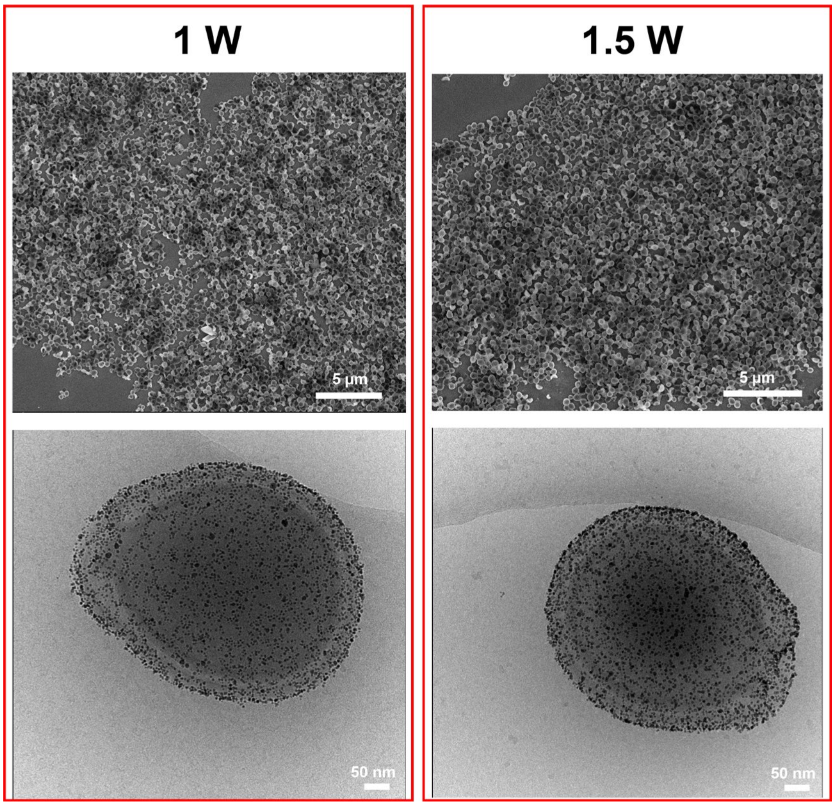

Supplementary Fig. 6. SEM (top) and cryo-TEM (down) images of Au-stomatocytes after irradiation with a 660 nm laser (1 W and 1.5 W) for 10 min, 3 experiments were repeated independently with similar results, scale bar (top) = 5 µm, (down) = 50 nm.

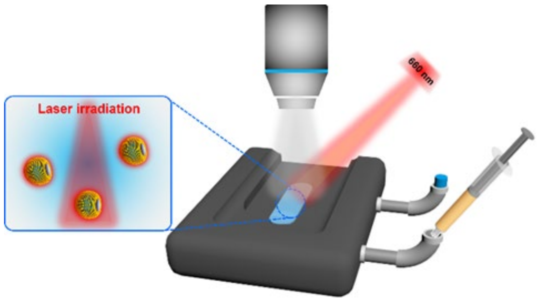

Supplementary Fig. 7. Schematic illustration of the experimental set-up for characterizing the motion of Au-stomatocytes using NanoSight equipped with an external 660 nm laser irradiation.

Table S5. Velocities of output laser power, corresponding with Fig 4e.

Output laser power (W)	Velocity (μm s^{-1})
0.75	19.5
1	47.7
1.1	73.3
1.2	91.2
1.3	95.4
1.4	104.5
1.5	124.7

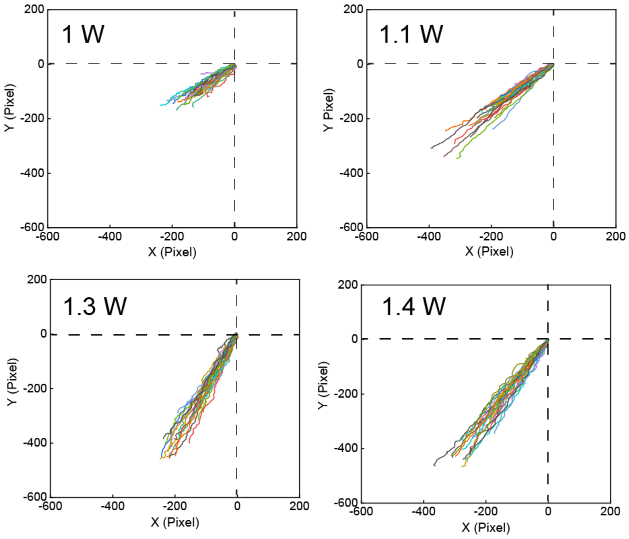

Supplementary Fig. 8. Motion trajectories of Au-stomatocyte nanomotors as a function of output laser power (1 W, 1.1 W, 1.3 W, and 1.4 W).

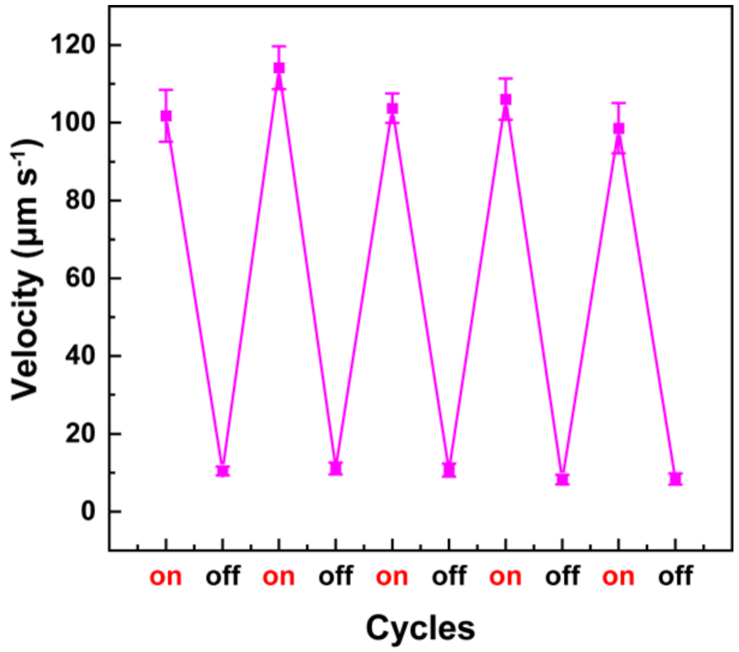

Supplementary Fig. 9. Velocity of Au-stomatocyte nanomotors in multiple on-off laser cycles (1.5 W).

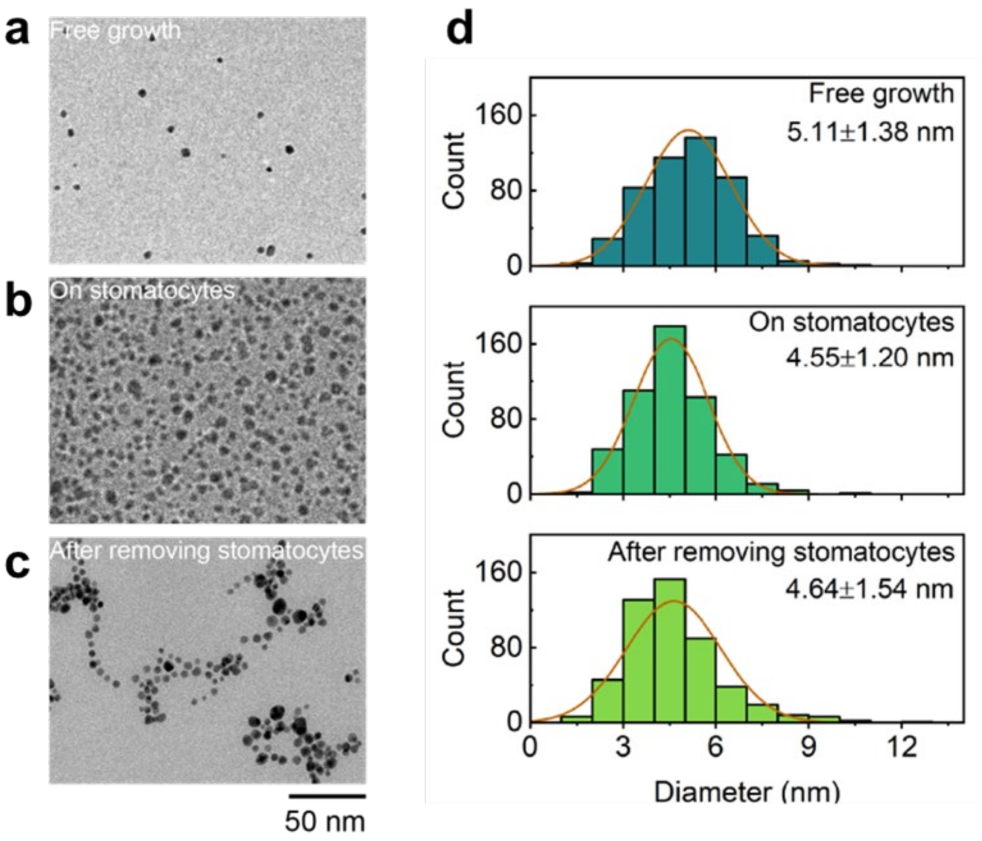

Supplementary Fig. 10. Statistical analysis of the size distribution of Au NPs grown with/without polymer stomatocytes. Cryo-TEM image of Au NPs grown **a)** freely in solution without stomatocytes (free growth) and **b)** on the stomatocytes. **c)** Dry-TEM of Au NPs that were collected from the stomatocytes, by removing the block copolymers with THF. 500 Au NPs were measured in each sample. **d)** Histograms showing the

size distribution of Au NPs from the above three samples. Interestingly, we found that Au NPs synthesized without stomatocytes were slightly larger than those grown on the surface of stomatocytes. This minor difference in size could be attributed to the fact that these freestanding Au NPs are not bound to a substrate and have a greater supply of Au precursors in the vicinity. All scale bars = 50 nm.

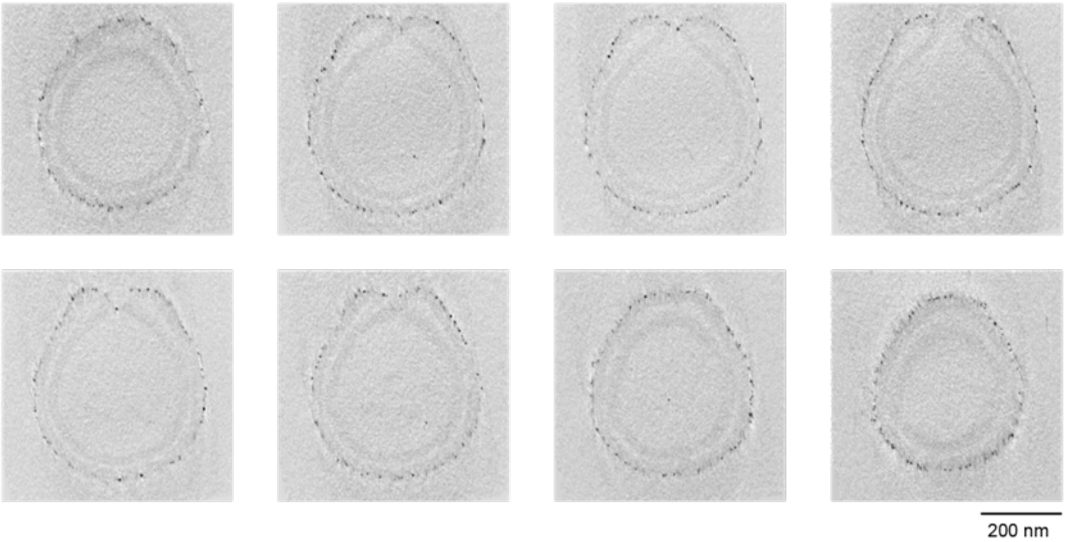

Supplementary Fig. 11. Representative *z*-slices from the 3D reconstruction of the Au-stomatocytes (Fig. 5) showing its cross-sectional morphology, 3 experiments were repeated independently with similar results, scale bar = 200 nm.

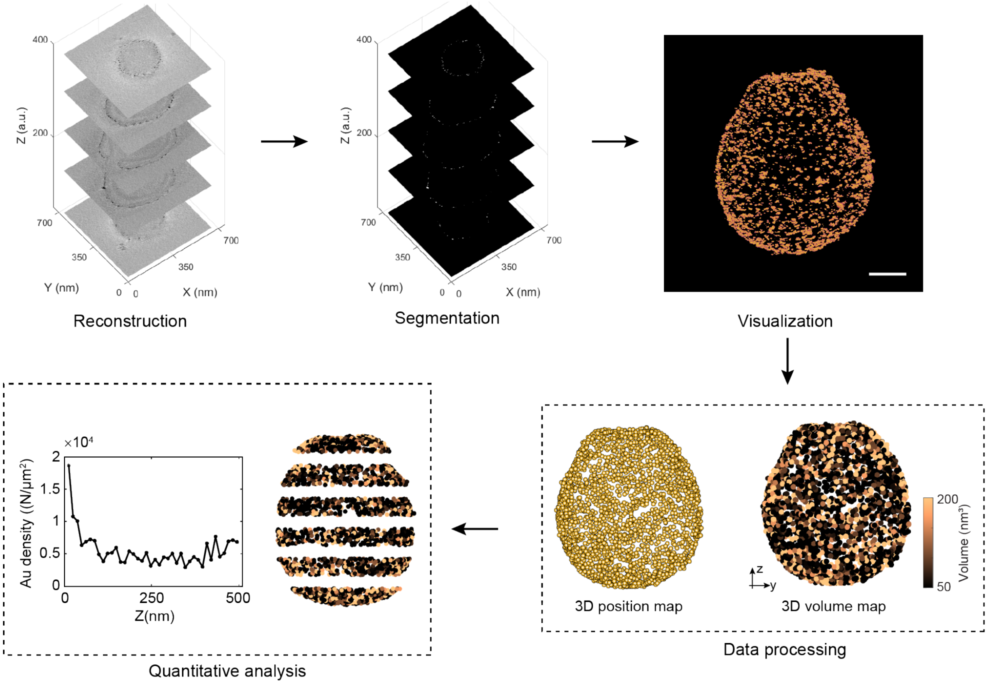

Supplementary Fig. 12. Image processing and analysis procedures of cryo-ET data. Scale bar = 100 nm.

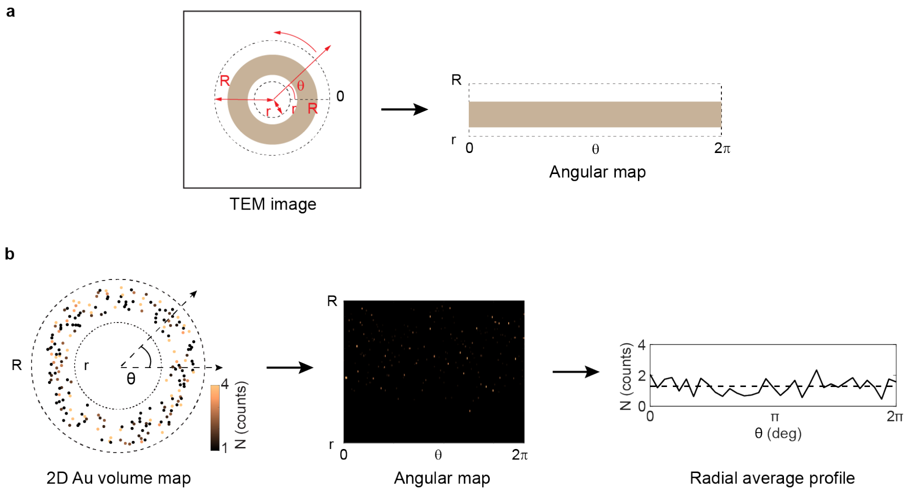

Supplementary Fig. 13. **a)** Schematic representation showing how to transform a 2D TEM image to the corresponding angular map.[28] **b)** Data analysis procedures to create a radial average profile from a 2D Au volume map at a certain z-height. The total intensity at each angle in the angular map is averaged across the radius to make a radial average profile.

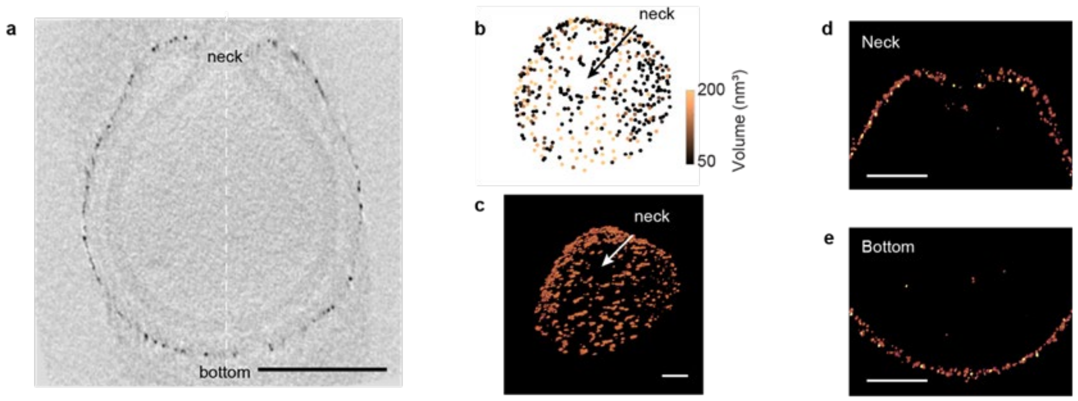

Supplementary Fig. 14. Distribution of Au nanoparticles around the neck and bottom of the stomatocyte. **a)** Central cross section of an Au stomatocyte from cryo-ET, where no Au nanoparticles are present in the narrowest part of the neck, 3 experiments were repeated independently with similar results. **b)** 3D position and volume map of Au nanoparticles in the neighborhood of the opening area. **c)** Volume rendering showing the cavity opening. **d-e)** Cross section showing the Au distribution in the area of the cavity opening and the stomatocyte bottom. Scale bars: **a)** 200 nm. **c-e)** 100 nm.

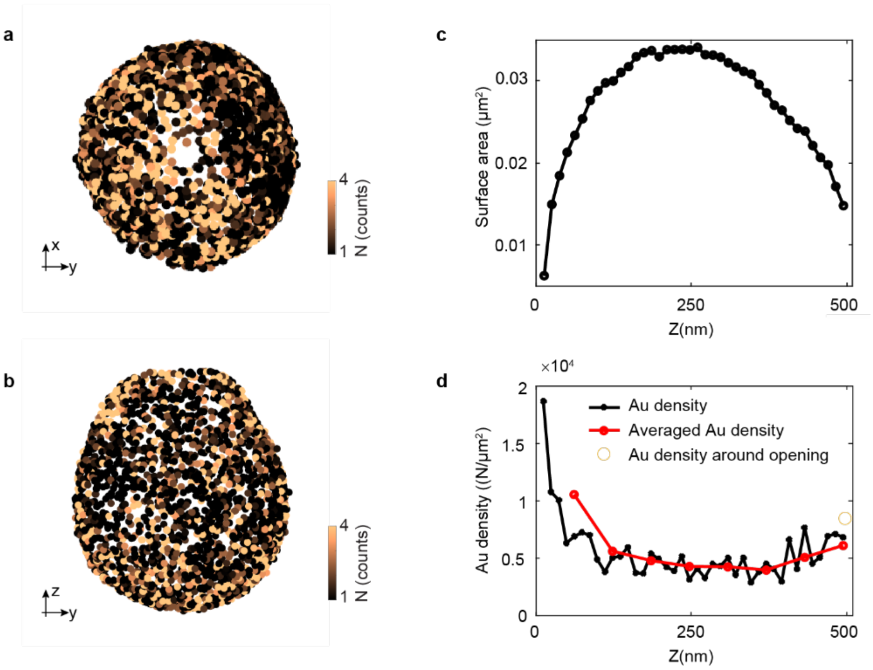

Supplementary Fig. 15. Quantification of lateral surface area and Au density changes along the z-axis. **a-b)** 3D Au distribution on the surface of a single Au-stomatocyte viewed from two different directions: top view (a) and side view **b)**. Note that the Au nanoparticles inside the cavity and on the top surface around the cavity opening are removed in **a)** and **b)**. **c)** Lateral surface area changes as a function of time. **d)** Au density changes as a function of time.

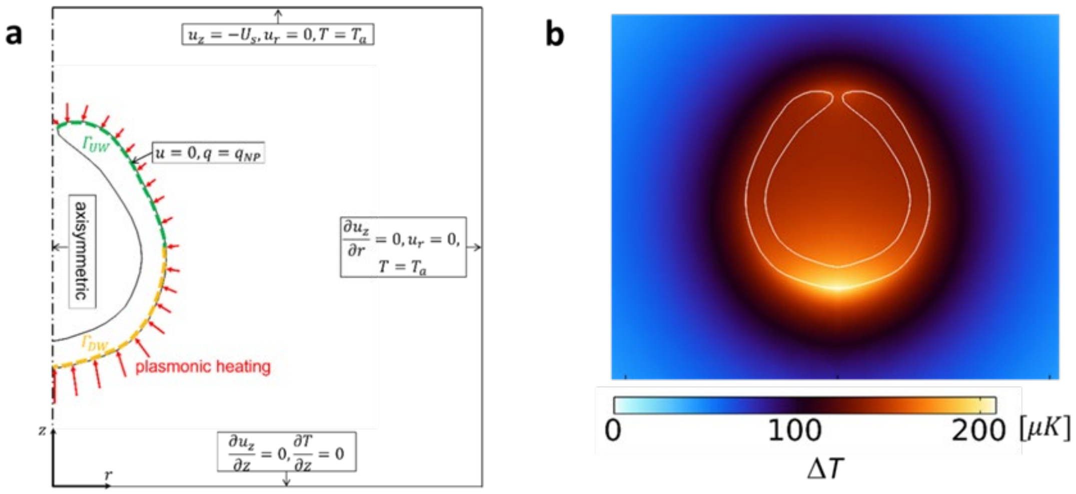

Supplementary Fig. 16. Understanding the temperature distribution around a single Au-stomatocyte using Finite Element Method (FEM) simulations. **a)** Numerical configuration for 2D axisymmetric simulation with the corresponding boundary conditions. Plasmonic heating of NPs is modeled as a surface heat flux distributed on the stomatocyte's surface. z and r represent the axial and radial coordinates, respectively. U_s represents the stomatocyte's swimming velocity measured in experiments. Γ_{UW} and Γ_{DW} denote the upwind and downwind surfaces of the stomatocyte. **b)** Simulated temperature field around a single Au-stomatocyte under 1.5 W laser irradiation. Note that here we show the temperature increase ΔT instead of the temperature.

Table S6 Overview of calculated surface averaged ∇T and drag forces at different laser output powers and velocities.

Output laser power (W)	Surface averaged ∇T (μK μm^{-1})	Velocity (μm s^{-1})	Drag force (× 10^{-14} N)
0.75	42.3	19.5	5.06
1.0	55.4	47.7	12.4
1.1	62.1	73.3	19.0
1.2	67.9	91.2	23.6
1.3	73.5	95.4	24.7
1.4	79.1	104.5	27.1
1.5	85.0	124.7	32.3

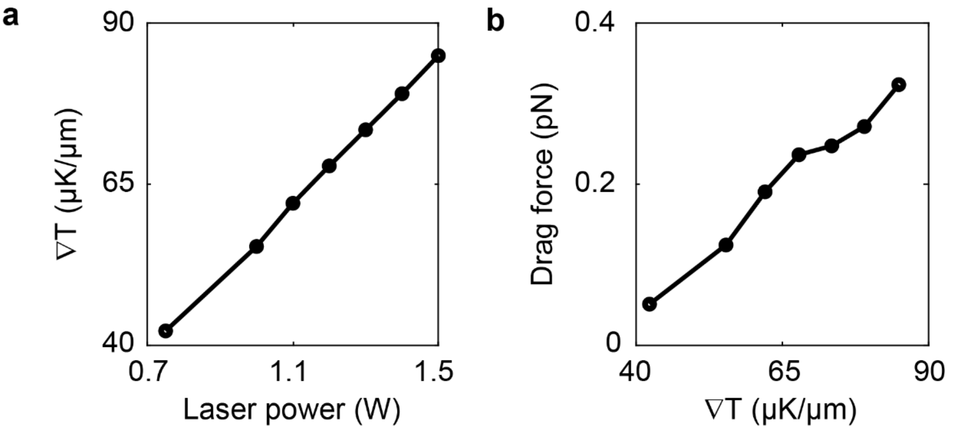

Supplementary Fig. 17. **a)** The measured averaged temperature gradient ∇T around a moving stomatocyte as a function of laser output power. **b)** The calculated drag force exerted on the stomatocyte as a function of ∇T.

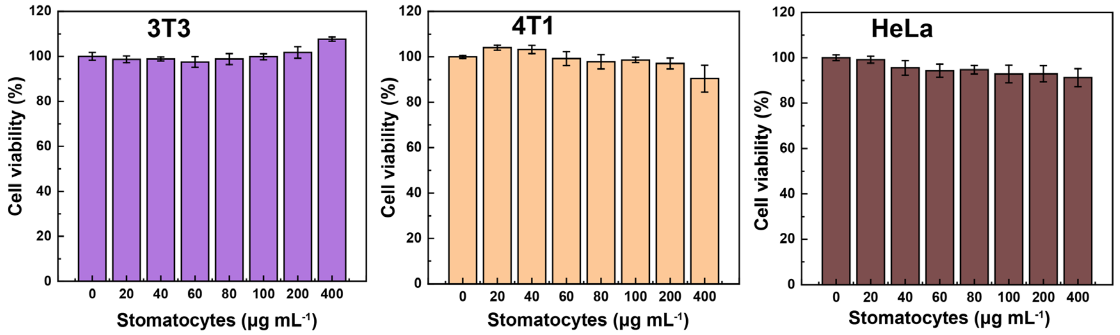

Supplementary Fig. 18. CCK-8 viability assay of 3T3, 4T1, and HeLa cells after incubation with Au-stomatocytes for 24 h. 3 experiments were repeated independently.

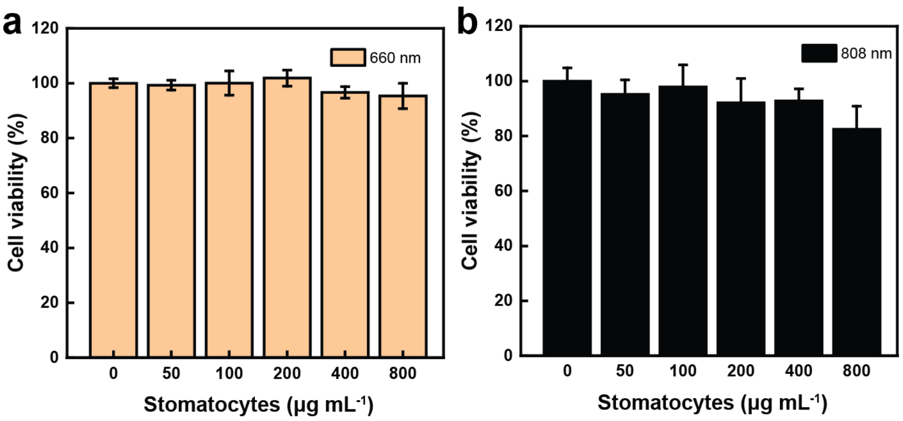

Supplementary Fig. 19. CCK-8 viability assay of HeLa cells after irradiation with **a)** a 660 nm laser and **b)** a 808 nm laser with 1 W output laser power for 5 min, followed by incubation for another 24 h. 3 experiments were repeated independently with similar results.

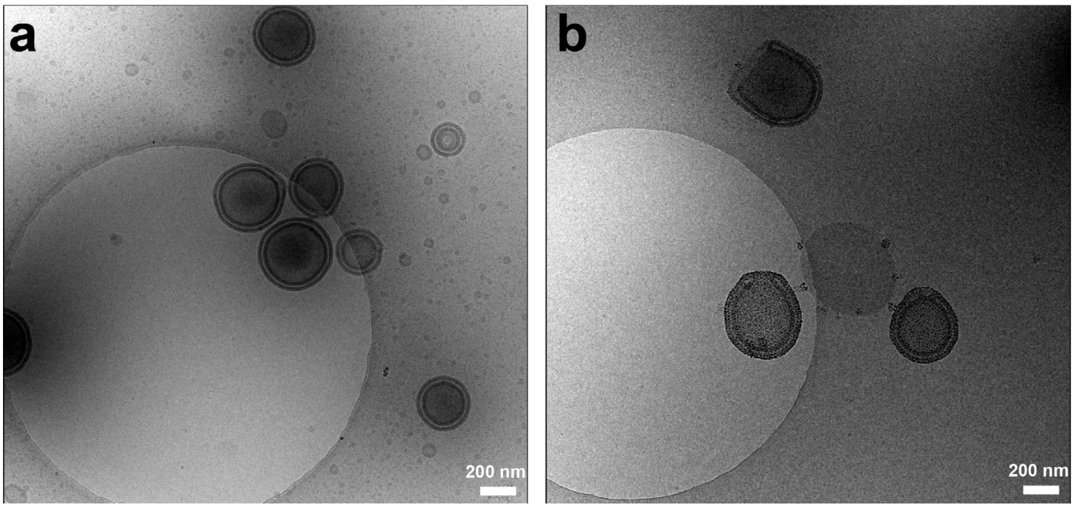

Supplementary Fig. 20. Cryo-TEM image of **a)** DOX loaded stomatocytes and **b)** DOX loaded Au-stomatocytes, 5 experiments were repeated independently with similar results. all the scale bars = 200 nm.

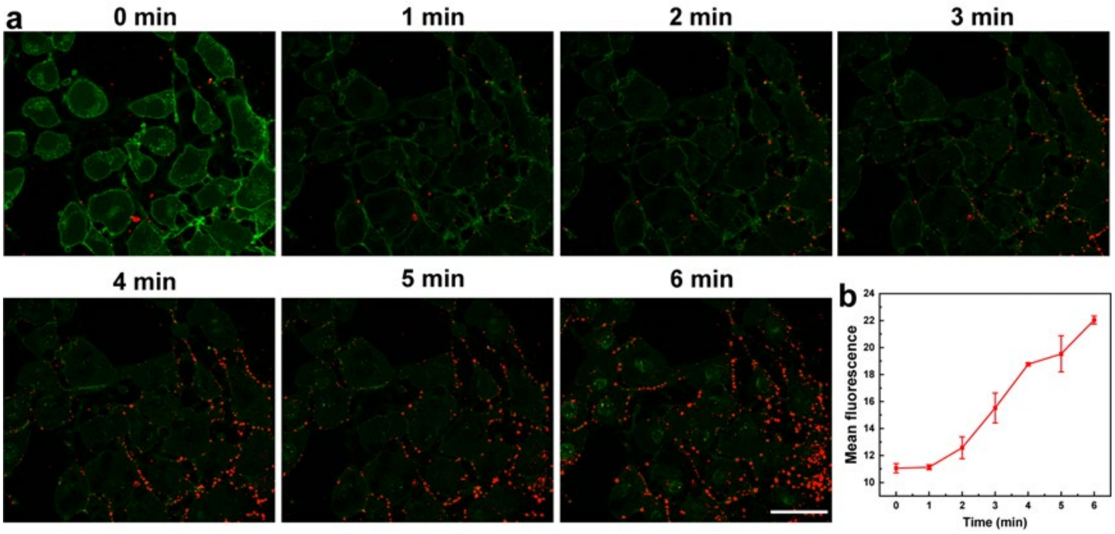

Supplementary Fig. 21. Enhanced cell uptake toward Au-stomatocytes upon laser irradiation. **a)** Time-lapsed CLSM images of the interaction between DOX loaded Au-stomatocytes and HeLa cells. The red fluorescence was from encapsulated DOX. Green fluorescence was from the cell membrane marker (WGA-AF488). 3 experiments were repeated independently with similar results. Scale bar = 50 μm. **b)** The mean fluorescence intensity of DOX analyzed by ImageJ.

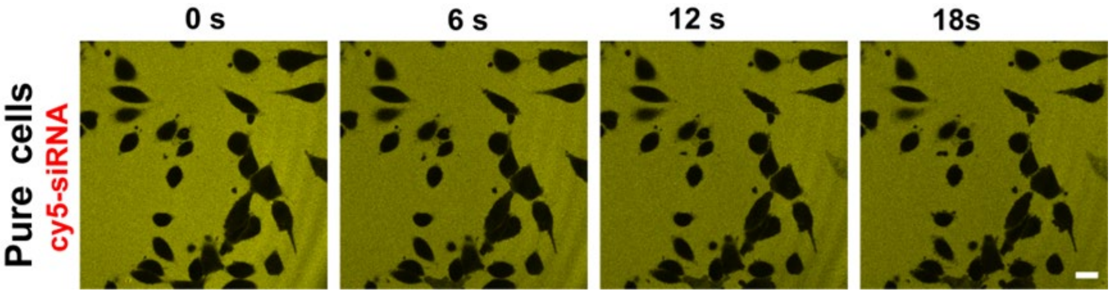

Supplementary Fig. 22. CLSM images of the transportation of cy5-siRNA into HeLa cells in the absence of Au-stomatocytes. Yellow fluorescence was from cy5-siRNA, 3 experiments were repeated independently with similar results. scale bar = 20 μm.

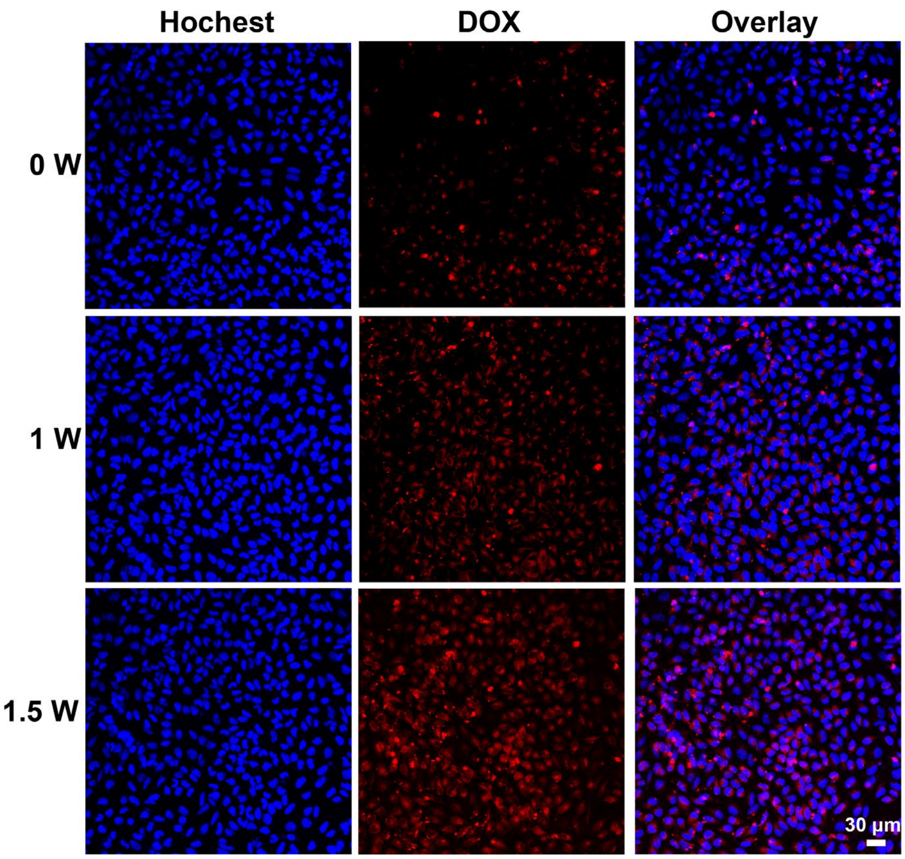

Supplementary Fig. 23. Confocal images of HeLa cells after taking up the DOX loaded Au-stomatocyte nanomotors ([DOX] = 2 μg mL^{-1}); the HeLa cells were immediately irradiated with an external 660 nm laser (output laser power: 0, 1, and 1.5W) after adding the Au-stomatocytes, followed by co-culturing for another 6 h. 3 experiments were repeated independently with similar results. Scale bar = 30 μm.

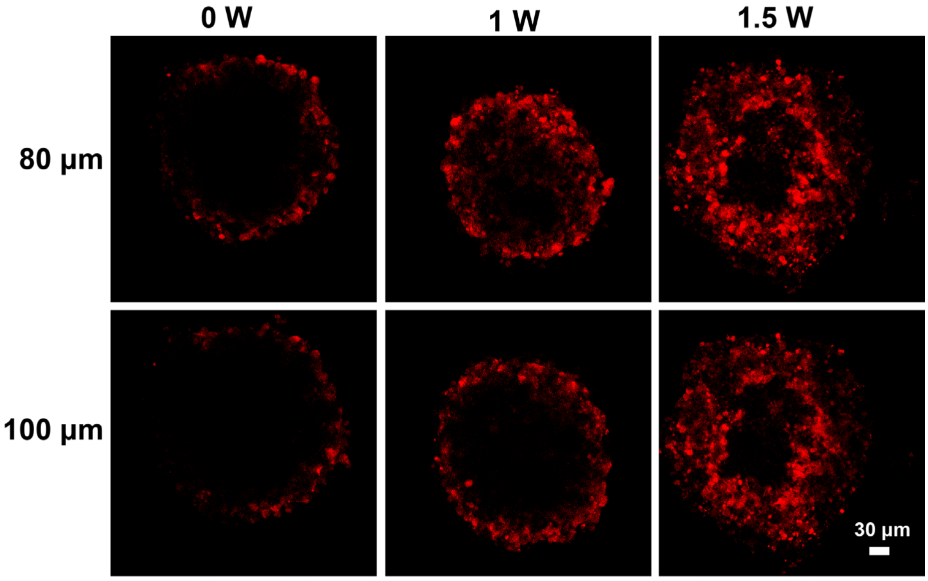

Supplementary Fig. 24. Representative z-scanning confocal images (80 and 100 μm) of HeLa 3D spheroids to investigate the tumor penetration of DOX loaded Au-stomatocytes ([DOX] = 2 μg mL^{-1}); the HeLa cells were immediately irradiated with an external 660 nm laser (output laser power: 0, 1, and 1.5W) after adding the Au-stomatocytes, followed by co-culturing for another 6 h. The surface of the tumor

spheroids was defined as 0 mm. 3 experiments were repeated independently with similar results. Scale bar = 30 μm.

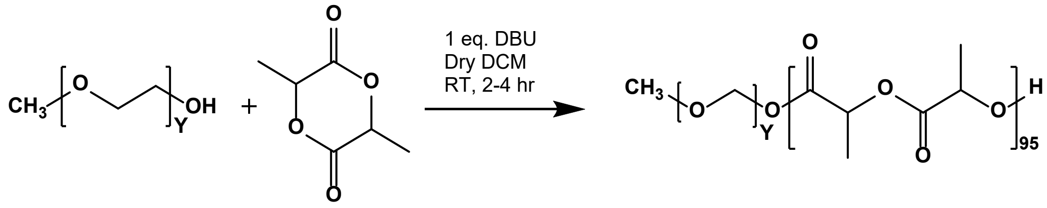

$PEG_{22/44}$ DLL $PEG_{22/44}$-$PDLLA_{95}$

Y = 22 or 44

Supplementary Fig. 25. Synthetic route of $PEG_{22/44}$-$PDLLA_{95}$ block polymers.

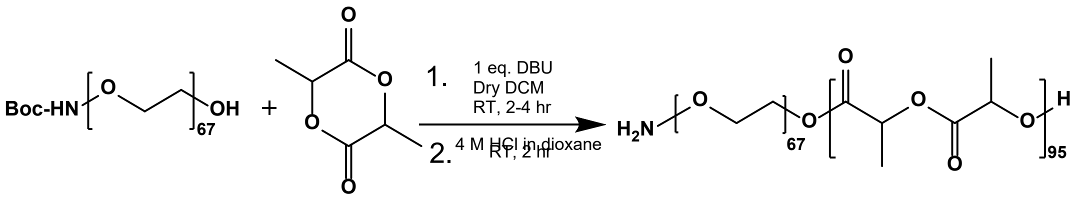

Boc-NH-PEG_{67} DLL NH_2-PEG_{67}-$PDLLA_{95}$

Supplementary Fig. 26. Synthetic route of amino terminated PEG_{67}-$PDLLA_{95}$ block polymer.

Supplementary references

1. Schindelin, J. et al. Fiji: an open-source platform for biological-image analysis. *Nat. Methods* **9**, 676-682 (2012).
2. Otsu, N. A Threshold Selection Method from Gray-Level Histograms. *IEEE Transactions on Systems Man and Cybernetics* **19**, 62-66 (1979).
3. Schwartz, J. et al. Real-time 3D analysis during electron tomography using tomviz. *Nat Commun.* **13**, 4458 (2022).
4. Yang, Y. et al. Deciphering chemical order/disorder and material properties at the single-atom level. *Nature* **542**, 75-79 (2017).
5. Cao, S. P. et al. Photoactivated nanomotors via aggregation induced emission for enhanced phototherapy. *Nat Commun.* **12**, 2077 (2021).
6. Pijpers, I. A. B. et al. Hybrid Biodegradable Nanomotors through Compartmentalized Synthesis. *Nano Lett.* **20**, 4472-4480 (2020).
7. Shao, J. X.; Cao, S. P.; Williams, D. S.; Abdelmohsen, L. K. E. A. & van Hest, J. C. M. Photoactivated Polymersome Nanomotors: Traversing Biological Barriers. *Angew Chem Int Ed.* **59**, 16918-16925 (2020).
8. Wauters, A. C. et al. Artificial Antigen-Presenting Cell Topology Dictates T Cell Activation. *ACS Nano* **16**, 15072–15085 (2022).
9. Zhou, M. Y. et al. Cancer Cell Membrane Camouflaged Semi-Yolk@Spiky-Shell Nanomotor for Enhanced Cell Adhesion and Synergistic Therapy. *Small* **16**, 2003834 (2020).
10. Gui, L. et al. Near-infrared light-driven multifunctional metal ion (Cu^{2+})-loaded polydopamine nanomotors for therapeutic angiogenesis in critical limb ischemia. *Nano Res.* **16**, 5108–5120 (2023).
11. Wang, W. J. et al. Chemical-NIR dual-powered CuS/Pt nanomotors for tumor hypoxia modulation,

deep tumor penetration and augmented synergistic phototherapy. *J. Mater. Sci. Technol.* **148**, 171-185 (2023).

12. Liu, M. H. et al. Light-driven Au-ZnO nanorod motors for enhanced photocatalytic degradation of tetracycline. *Nanoscale* **14**, 12804-12813 (2022).
13. Xuan, M. J. et al. Near Infrared Light-Powered Janus Mesoporous Silica Nanoparticle Motors. *J. Am. Chem. Soc***.** **138**, 6492-6497 (2016).
14. Wang, Y. F. et al. NIR-II Light Powered Asymmetric Hydrogel Nanomotors for Enhanced Immunochemotherapy. *Angew. Chem. Int. Ed.* **62**, e202212866 (2022).
15. Zhou, D. K. et al. Light-Ultrasound Driven Collective "Firework" Behavior of Nanomotors. *Adv. Sci.* **5**, 1800122 (2018).
16. Maric, T.; Lovind, A.; Zhang, Z. Y.; Geng, J. Y. & Boisen, A. Near-Infrared Light-Driven Mesoporous SiO2/Au Nanomotors for Eradication of Pseudomonas aeruginosa Biofilm. *Adv. Healthc. Mater.* **12**, 2203018 (2023).
17. Yu, J. Y. et al. Self-Propelled Enzymatic Nanomotors from Prodrug-Skeletal Zeolitic Imidazolate Frameworks for Boosting Multimodel Cancer Therapy Efficiency. *Adv. Sci***.** **10**, 2301919 (2023).
18. Teo, W. Z.; Zboril, R.; Medrik, I. & Pumera, M. Fe-0 Nanomotors in Ton Quantities (10(20) Units) for Environmental Remediation. *Chem-Eur. J***.** **22**, 4789-4793 (2016).
19. Zheng, J. et al. Cascade Catalytically Released Nitric Oxide-Driven Nanomotor with Enhanced Penetration for Antibiofilm. *Small* **18**, 2205252 (2022).
20. Tang, X. T. et al. Lipophilic NO-Driven Nanomotors as Drug Balloon Coating for the Treatment of Atherosclerosis. *Small* **19**, 2203238 (2022).
21. Fu, D. M.; Ye, Y. C.; Gao, C.; Xie, D. Z. & Peng, F. Bienzymatic Spiky Janus Nanomotors Powered by Histamine. *ChemNanoMat* **8**, e20220015 (2022).
22. Cao, S. P. et al. Cucurbit-Like Polymersomes with Aggregation-Induced Emission Properties Show Enzyme-Mediated Motility. *ACS Nano* **15**, 18270-18278 (2021).
23. Ou, J. F. et al. MnO_2-Based Nanomotors with Active Fenton-like Mn^{2+} Delivery for Enhanced Chemodynamic Therapy. *ACS Appl. Mater. Inter***.** **13** , 38050-38060 (2021).
24. Ma, Y. Z. et al. Streamlined Mesoporous Silica Nanoparticles with Tunable Curvature from Interfacial Dynamic-Migration Strategy for Nanomotors. *Nano Lett.* **21**, 6071-6079 (2021).
25. Kwon, T. et al. Au/Pt-Egg-in-Nest Nanomotor for Glucose-Powered Catalytic Motion and Enhanced Molecular Transport to Living Cells. *Angew. Chem. Int. Ed.* **60**, 17579-17586 (2021).
26. Zhou, C. et al. Autonomous Motion of Bubble-Powered Carbonaceous Nanoflask Motors. *Langmuir* **36**, 7039-7045 (2020).
27. Choi, H.; Cho, S. H. & Hahn, S. K. Urease-Powered Polydopamine Nanomotors for Intravesical Therapy of Bladder Diseases. *ACS Nano* **14**, 6683-6692 (2020).
28. Ianiro, A. et al. Liquid-liquid phase separation during amphiphilic self-assembly. *Nat. Chem.* **11**, 320-328 (2019).
