# Supplementary material for: Ultrafast light-activated polymeric nanomotors
Source: Nat Commun. 2024 Jun 7;15:4878. doi: 10.1038/s41467-024-49217-w (PMC11161643; doi:10.1038/s41467-024-49217-w)
Supplement: Supplementary file 3 — Description of Additional Supplementary Files [file 41467_2024_49217_MOESM3_ESM.pdf]

Description of Additional Supplementary Files

Supplementary Movie Legends

Supplementary Movie 1. Nanoparticle tracking video of Au-polymersomes under laser irradiation (660 nm, 1 W).

Supplementary Movie 2. Nanoparticle tracking video of Au-stomatocytes under laser irradiation (660 nm, 1 W)

Supplementary Movie 3. Nanoparticle tracking video of Au-stomatocytes without laser irradiation (660 nm, 0 W)

Supplementary Movie 4. Motion behavior of Au-stomatocytes under laser irradiation (660 nm, 1.5 W)

Supplementary Movie 5. Motion behavior of Au-stomatocytes under laser irradiation from bottom right to the upper left (660 nm, 1.5 W)

Supplementary Movie 6. Cryo-electron tomography 3D reconstruction of a typical Au-stomatocyte. Playback frame rate: 50 fps.

Supplementary Movie 7. Nanoparticle tracking video of DOX-stomatocytes without TP-NIR irradiation

Supplementary Movie 8. Motion behavior of DOX-stomatocytes with TP-NIR irradiation
